# Analysis of Propagation and Distribution Characteristics of Leakage Acoustic Waves in Water Supply Pipelines

**DOI:** 10.3390/s21165450

**Published:** 2021-08-12

**Authors:** Yunfei Li, Yang Zhou, Ming Fu, Fan Zhou, Zhaozhao Chi, Weihao Wang

**Affiliations:** 1 Hefei Institute for Public Safety Research, Tsinghua University, Hefei 230601, China; lyf020@mail.ustc.edu.cn (Y.L.); zhouyang@gsafety.com (Y.Z.); zhoufan@gsafety.com (F.Z.); chizhaozhao@gsafety.com (Z.C.); wangweihao@tsinghua-hf.edu.cn (W.W.); 2 Key Laboratory of Personnel Safety in Disaster Environment, Hefei 230601, China

**Keywords:** water supply pipeline, leakage acoustic wave, propagation in pipeline, distribution characteristics, spherical detector

## Abstract

Leakage detection methods based on the analysis of leakage acoustic signals provide an effective technical approach for detecting small leaks in water supply pipelines. From a technical perspective, the study of the propagation characteristics of acoustic waves generated by the leakage in the water supply pipeline is necessary for detecting the leak location on the basis of acoustic signals. In this study, a 3D transient leakage acoustic wave propagation equation was derived by combining the principles of fluid dynamics and Lighthill acoustic analogy theory. The propagation of the leakage-induced noise in water supply pipeline was modelled theoretically. We simulated the propagation of a leakage acoustic wave under different conditions for different target scenarios encountered in actual pipeline inspections. Specifically, we analysed the effect of different factors, such as the pipe size and acoustic source characteristics, on acoustic propagation. Finally, the simulated experiments were practically performed using a self-designed simulated water supply pipeline and self-developed spherical water supply pipeline detector to validate the simulation analysis. The results of this study provide a theoretical guidance and basis for the analysis of characteristics of leakage acoustic wave signals and the recognition of leakage conditions in water supply pipelines.

## 1. Introduction

The increasing level of urbanisation has led to an increase in the scale of water supply pipelines constructed in urban areas [1]. Owing to obsolete water supply infrastructures, complex maintenance operations in densely populated areas, and high maintenance costs, the leakage rate of water supply networks has reached 25% [2]. Leaks in water supply pipelines cause not only economic losses but also large-scale water road collapses and other accidents [3,4]. Therefore, it is very important to detect leakage in water supply pipelines [5].

Listening to the leak sounds at the surface of the pipeline is currently the most commonly used method for water leakage detection [6,7,8]. However, this method is not suitable for the accurate detection of small leaks over a long distance owing to the limited effective detection distance and complicated background noise in the external environment of the pipeline. The mobile leak detection method, which is based on the acoustic signal, is an effective approach to overcome the issues of traditional leakage detection methods [9,10]. From a technical perspective, to detect the leak location using this method, it is necessary to study the propagation characteristics of acoustic waves generated by the leakage in the water supply pipeline.

The generation mechanism and propagation characteristics of leak acoustic signals in pipelines have been investigated by many researchers worldwide. Liu et al. [11] found that quadrupole and dipole sonic sources are the major causes of leak-acoustics in gas pipelines. They analysed the effect of the leakage orifice diameter on the pressure perturbation generated by quadrupoles and dipoles. Kostowski et al. [12] simulated the steady-state flow of liquids in pipelines by using a one-dimensional isothermal and adiabatic flow model. Further, they proposed a method to account for the leakage by means of a reference flow equation with a discharge coefficient.

With the increasing depth of research on leakage detection, the propagation model of leak acoustic signals has gradually evolved from one-dimensional to three-dimensional. Han et al. [13] studied the generation mechanism of acoustic sources in gas pipeline leakage and then developed a simulation model based on the aeroacoustics, flow field, and sound source characteristics of the gas pipeline leakage. Yuan et al. [14] performed a three-dimensional computational fluid dynamic study on the generation and propagation mechanism of negative waves in high-pressure gas pipelines using the large eddy simulation approach. They proposed a method to predict the negative wave amplitude on the basis of the pipeline pressure and leak diameter. Xu et al. [15] analysed the propagation of leak acoustic signals along the wall of a gas pipeline theoretically. On the basis of this analysis, they further identified the acoustic signal of gas pipeline leakage by using the wavelet packet transform method and fuzzy support vector machine pattern classification.

Masri et al. [16] investigated leakage from a pipeline with an incompressible flow. They introduced continuous sinusoidal pressure waves of small amplitudes at the entrance of the pipeline and simulated the variation of the amplitude of the pressure gradient and the shear stress on the inside surface of the pipeline in the streamwise flow direction. For a water supply pipeline, Yan et al. [17] analysed the soil–pipe–liquid three-phase coupling problem associated with buried pipelines. By treating the soil around the pipeline as an elastic body, they studied the acoustic propagation characteristic in a liquid-filled pipeline surrounded by an elastic medium. Yang et al. [18] explored the propagation and attenuation characteristics of the leak acoustic wave along the direction of the water flow and further detected and analysed the leak acoustic wave signal in the water supply pipeline. Giustolisi et al. [19] proposed a novel steady-state simulation model of a water supply network considering the classical hydraulic representation, pressure-driven demand, and leakage at the pipeline level. The experimental tests demonstrated a high robustness of the proposed simulation model. Lu et al. [20] treated the water supply pipeline as the signal transmission system and studied the dispersion characteristics of leakage acoustic waves of the water supply pipeline during wave propagation. Abhulimen et al. [21] derived a novel model for detecting leaks in complex pipeline network systems based on the Liapunov stability criteria. Further, they analysed the unsteady state flow matrix of the complex pipeline network system to compute the velocity and pressure for each node and pipeline loop in the complex network.

All the aforementioned studies have focused on the generation mechanism and propagation characteristics of leakage acoustic waves in pipelines. These studies provided a theoretical basis for improving the methods and techniques used to detect pipeline leakage. However, most existing studies emphasise on the propagation mechanism and the characteristics of leak acoustic waves at the leaking location and along the pipeline wall, particularly the propagation behaviour of leak acoustic waves along the medium in the pipeline. There are just a few studies on the distribution characteristics of leak acoustic waves. Therefore, to address this gap, this study aims to determine the technical requirements of leak acoustics detection in water supply pipelines. On the basis of the three-dimensional transient leakage acoustic propagation model in the pipeline, a theoretical model was developed to investigate the propagation of leak acoustic waves in the water supply pipeline. The variation in the sound pressure, sound intensity, and frequency distribution in the pipeline were analysed through simulation analysis and experiments. Moreover, the focus of current research has shifted to underground pipeline and pipeline network, and it is a Gordian knot for leakage detection. The conclusions from this study are mainly aimed at providing a fundamental theory for a spherical detector, which can be utilised in underground pipeline and pipe networks. With respect to other detecting methods, this study can also provide a theoretical guidance and basis for the analysis of the characteristics of leakage acoustic wave signals and the recognition of leakage conditions in water supply pipelines.

## 2. Theoretical Analysis

The acoustic mechanism of leakage acoustic waves generated in pressure pipelines can be rationalised based on aeroacoustics [22]. The mechanism of acoustic wave generation is essential in fluid–fluid and fluid–solid interactions. Therefore, the distribution of the acoustic sources in the flow field in water supply pipelines can be obtained from the fundamental equations of fluid dynamics, i.e., the Navier–Stokes equation (N–S equation) given by Equation (1) as follows:

(1) $$\rho \frac{\partial v}{\partial t}=\rho F-\nabla p+\mu \nabla^{2}v-\left(\nabla v\right)v,$$

where *ρ* is liquid density, *v* is the velocity vector, *p* is pressure, *F* is the external force scalar, and *μ* is the dynamic viscosity.

Noise generation, due to the leakage in water supply pipelines, is a complex fluid–solid coupling process. Owing to the large pressure difference between the internal and external sides of the pipeline wall, the leakage location becomes a pressure outlet with a small cross-sectional area. Thus, the large pressure difference leads to the formation of a high-velocity jet stream at the leaking point. Furthermore, the irregular geometry of the leakage results in complex fluid–solid coupling vibration and the formation of turbulent flow. The pulsation of the turbulent flow generates fluid noise, which propagates in the pipeline with the contained liquid as a propagation medium. This acoustic source formed by high-speed pulsating turbulence generated by high pressure is also called the quadrupole source of sound radiation. The propagation of sound in a uniform medium, without sources of matter or external forces, is governed by the following equations (Lighthill et al.) [23]:

(2) $$\frac{\partial \rho }{\partial t}+\frac{\partial }{\partial x_{i}}\left(\rho v_{i}\right)=0$$

(3) $$\frac{\partial }{\partial t}\left(\rho v_{i}\right)+a_{0}^{2}\frac{\partial \rho }{\partial x_{i}}=0$$

(4) $$\frac{\partial^{2}\rho }{\partial t^{2}}-a_{0}^{2}\nabla^{2}\rho =0$$

where $v_{i}$ is the velocity in the $x_{i}$ direction and $a_{0}$ is the speed of sound in the uniform medium. Equation (4) is attained from eliminating the momentum density $\rho v_{i}$ from Equations (2) and (3).

Specifically, the wave equation of turbulent noise can be derived by re-writing the two sides of the N–S equation in the wave form as [19]. Substitute $p=a_{0}^{2}\left(\rho -\rho_{0}\right)$ and Equation (4) into Equation (1) as follows:

(5) $$\frac{\partial^{2}\rho }{\partial t^{2}}-\frac{\partial^{2}\rho }{\partial x_{i}{}^{2}}=\frac{\partial^{2}T_{ij}}{\partial x_{i}x_{j}},$$

(6) $$T_{ij}=\rho u_{i}u_{j}+\left[\left(p-p_{0}\right)-\left(\rho -\rho_{0}\right)\right]\delta_{ij},$$

(7) $$\left\{\begin{array}{l} \delta_{ij}=1,i=j\\ \delta_{ij}=0,i\neq j \end{array}\right..$$

In these equations, *T* is the Lighthill stress tensor, *ρ* is the liquid density, *ρ*_0_ is the density of free liquid, *t* is the time; *c* is the speed of sound in the uniform medium, *δ_ij_* is the Kronecker delta, *u_i_* and *u_j_* are the velocity components, *p* is the fluid pressure, *p*_0_ is the static pressure of the flow field, $\partial^{2}T_{ij}/\partial x_{i}x_{j}$ is the Lighthill quadrupole acoustic source, and *T_ij_* is the element in the turbulence stress tensor matrix. If all the components in the stress tensor equal to zero, then no acoustic waves are generated. Once a leakage occurs in the water supply pipeline, a high-velocity jet stream at the leak location must exist. Therefore, *ρv_i_v_j_* is a finite and non-zero limited term, and an acoustic wave is inevitably generated.

## 3. Model Construction

The leak acoustic model in the pipeline can be constructed using the classical wave equation of fluids. The leakage is assumed to generate an adiabatic noise with a small amplitude, and the flow is assumed to be uniform in the pipeline. When the flow velocity is much smaller than the local Mach number, the fluid velocity has a negligible impact on acoustic propagation in the liquid. Therefore, the influence of the flow velocity on acoustic propagation is neglected in this study.

**Figure 1:**
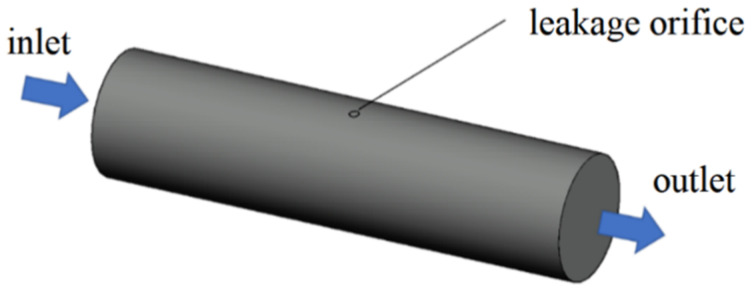
Geometric model of leakage in the pipeline.

The diameter of urban underground water supply pipelines varies from 100 to 1200 mm according to the different levels of water consumption in the local area. The primary urban underground water supply pipelines have diameters of 500 mm. Figure 1 shows the geometric model of leakage from a pipeline. The section used for our computation has a length of 20 m. To investigate the effect of the diameter on the propagation of leakage acoustic signals in the water supply pipeline, two different diameters (i.e., 200 and 500 mm) were considered in this study. A uniform velocity profile of 1 m/s was used as the boundary condition at the inlet. A zero-pressure boundary condition was imposed on the outlet. The acoustic source at the leakage location was considered as a point acoustic source located at the centre of the pipeline.

A three-dimensional transient acoustic wave equation was derived in the cylindrical coordinate based on the Lighthill turbulent noise wave equation, as expressed by Equation (5). In this model, the amplitude of the sound wave is small. For the convenience of the model calculation, $\frac{\partial^{2}T_{ij}}{\partial x_{i}x_{j}}$ is ignored in Equation (5). Change Equation (5) from linear to three-dimensional, as expressed by Equation (8) [23] as follows:

(8) $$\nabla^{2}p=\frac{1}{r}\frac{\partial }{\partial r}\left(r\frac{\partial p}{\partial r}\right)+\frac{1}{r^{2}}\frac{\partial^{2}p}{\partial \varphi^{2}}+\frac{\partial^{2}p}{\partial z^{2}}=\frac{1}{a_{0}^{2}}\frac{\partial^{2}p}{\partial t^{2}},$$

where *r*, the radial coordinate, equals to *x_j_*; *φ* is the azimuthal angle; *z*, the coordinate along the axial direction of the pipeline, equals to *x_i_*; *p* is the acoustic pressure; *t* is the time; and $a_{0}$ is the speed of sound in water, which is 1497 m/s.

Considering that the flow is uniform, Equation (8) can be transformed to Equation (9) as follows:

(9) $$\frac{1}{r}\frac{\partial^{2}p}{\partial r^{2}}+\frac{1}{r^{2}}\frac{\partial^{2}p}{\partial \varphi^{2}}+\left(1-\frac{U^{2}}{a_{0}^{2}}\right)\frac{\partial^{2}p}{\partial z^{2}}=\frac{1}{a_{0}^{2}}\frac{\partial^{2}p}{\partial t^{2}}+2U\frac{1}{a_{0}^{2}}\frac{\partial^{2}p}{\partial z\partial t},$$

where *U* is the average flow velocity. In terms of the acoustic boundary condition, the pipeline wall was set as a hard boundary (total reflection of the acoustic wave without any absorption), which can be expressed by Equation (10) as follows:

(10) $$\frac{\partial p}{\partial r}=0.$$

A soft boundary condition (total absorption of the acoustic wave without any reflection) with zero acoustic pressure was imposed on the velocity inlet and the pressure outlet. As Equation (10) is linear, the superposition principle of the acoustic pressure is satisfied. Therefore, the point acoustic source is set as the superposition of multiple sine wave signals with different frequencies that change over time. The amplitude of these waves was set as 1, and the frequency varied from 1 to 20,000 Hz. The wave equation is shown as Equation (11). Considering that the high-frequency acoustic waves decay faster and possess less energy, the acoustic waves with a frequency exceeding 20 kHz were neglected in the simulation analysis.

(11) $$p=\overset{20,000}{\underset{f=1}{\sum }}\sin\left(2\pi ft\right).$$

Equation (11) is discretised with second-order precision. The second-order partial derivative term is discretised using the second-order central difference scheme, given by Equation (12).

(12) $$\frac{p_{i+1,j,z}^{n+1}-2p_{i,j,z}^{n+1}+p_{i-1,j,z}^{n+1}}{\Delta r^{2}}+\frac{1}{r}\frac{p_{i+1,j,z}^{n+1}-p_{i-1,j,z}^{n+1}}{2\Delta r}+\frac{1}{r^{2}}\frac{p_{i,j+1,z}^{n+1}-2p_{i,j,z}^{n+1}+p_{i,j-1,z}^{n+1}}{\Delta \varphi^{2}}+\;\left(1-\frac{U^{2}}{a_{0}^{2}}\right)\frac{p_{i,j,z+1}^{n+1}-2p_{i,j,z}^{n+1}+p_{i,j,z-1}^{n+1}}{\Delta x^{2}}=\frac{1}{a_{0}^{2}}\frac{p_{i,j,z}^{n+1}-2p_{i,j,z}^{n}+p_{i,j,z}^{n-1}}{\Delta t^{2}}+\frac{2U}{a_{0}^{2}}(\frac{p_{i,j,z+1}^{n+1}-p_{i,j,z+1}^{n}}{\Delta x\Delta t}-\frac{p_{i,j,z}^{n+1}-p_{i,j,z}^{n}}{\Delta x\Delta t})$$

The pipeline was divided into 100, 20, and 50 computation nodes along the axial, radial, and circumferential directions, respectively. Considering that the maximum frequency of the signal at the acoustic source is 20,000 Hz, and the corresponding period is 5 × 10^−5^ s, the timestep was set to 10^−7^ s to capture the wave characteristics of the acoustic source in the computation and improve the calculation accuracy. As the computational model developed in this study is a transient model, the distributions of the acoustic pressure and intensity were calculated at a total computational time of 0.1 s. In this case, the acoustic signals can propagate over a sufficiently long distance in the pipeline.

The discretised wave equation was solved considering the corresponding boundary conditions via the iteration method using a global convergence algorithm.

## 4. Simulation Analysis

### 4.1. Distribution of Acoustic Pressure in the Pipeline and Characteristics of Acoustic Decay

**Figure 2:**
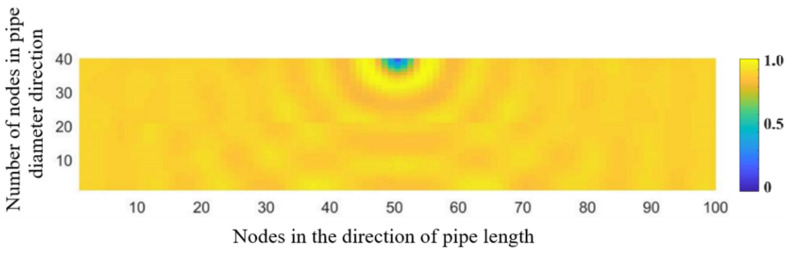
Distribution of acoustic pressure generated from a point-shaped leakage hole along the longitudinal cross section of the 20-meter-long pipeline.

**Figure 3:**
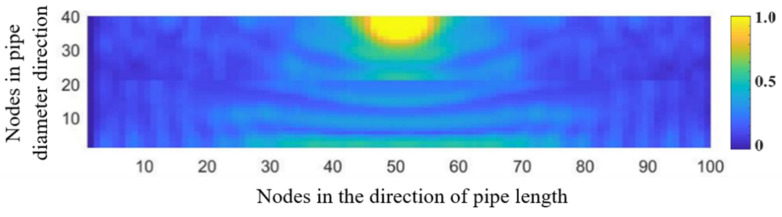
Distribution of the maximum amplitude of the acoustic pressure generated from a point-shaped leakage hole along the longitudinal cross section of the 20-meter-long pipeline.

Figure 2 shows the distribution of the acoustic wave along the longitudinal cross section of the pipeline. The propagation of the spherical wave and the reflection of the wave at the interface of the pipeline can be clearly seen. The acoustic waves reflected by the lower wall of the pipeline and those transmitted from the acoustic source are superimposed in the centre of the pipeline to form interference fringes. Figure 3 shows the distribution of the maximum amplitude of the acoustic pressure in the longitudinal cross section of the pipeline. The maximum amplitude reflects the intensity of the acoustic field in the pipeline. It is found that the acoustic intensity is quite strong near the acoustic source in the pipeline and decays very rapidly with increasing distance from the source. The region with a strong acoustic field is represented by a fan-shaped area formed at a certain angle and centred on the acoustic source. In addition, the distribution of the acoustic intensity follows a stripe shape in the pipeline. This behaviour is attributed to the interference between the reflected and mainstream waves, which produces a standing wave at a certain location in the pipeline.

**Figure 4:**
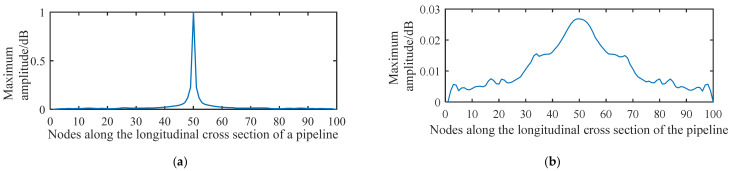

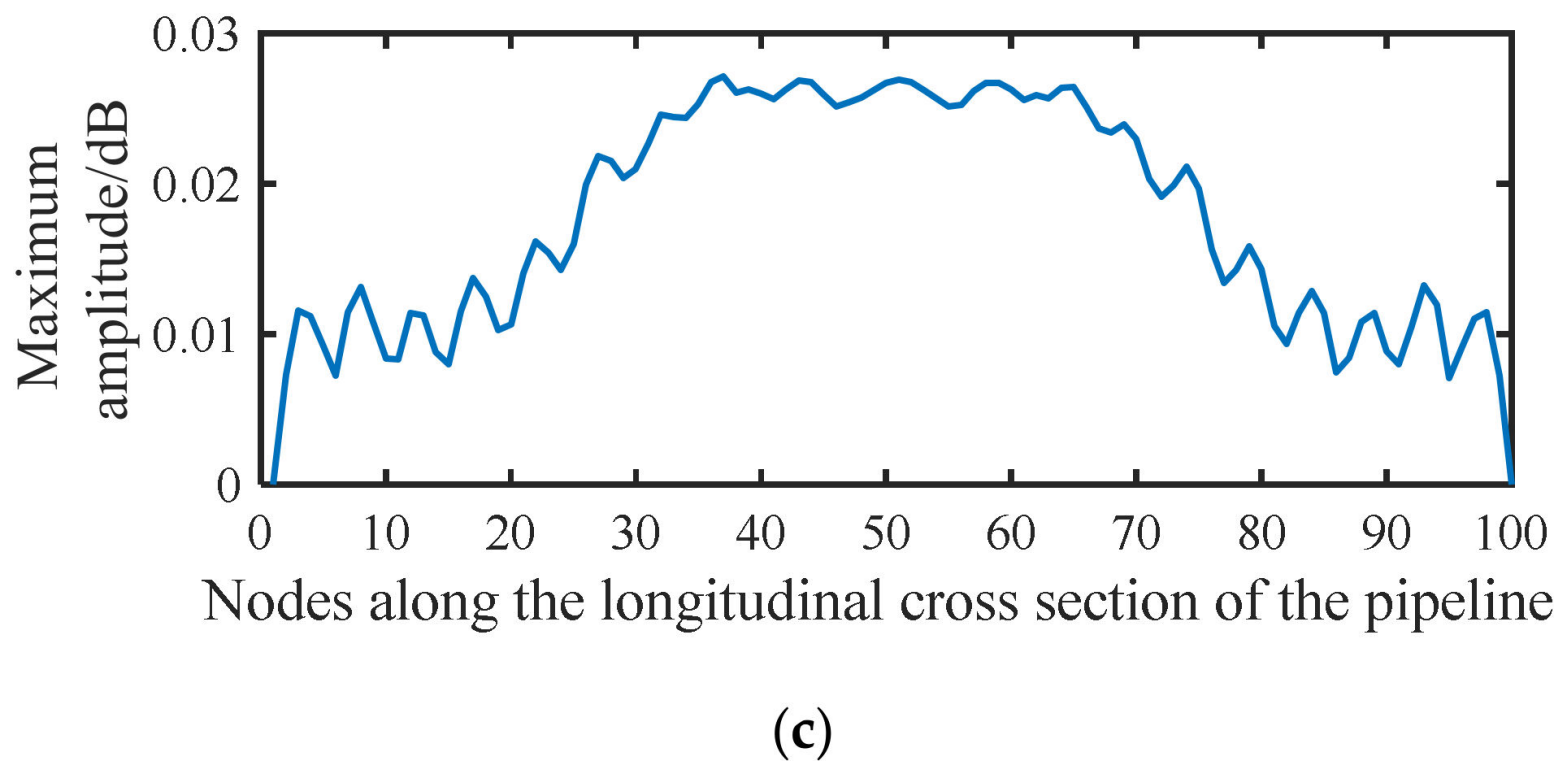
Distribution of the maximum amplitude of the acoustic pressure generated from a point-shaped leakage hole along the longitudinal cross section of the pipeline (**a**) nearest to the acoustic source; (**b**) on the central axis of the pipeline, and (**c**) far away from the acoustic source.

Figure 4 shows the distribution of the maximum amplitude of the acoustic pressure at three locations along the axial direction of the pipeline measured based on the longitudinal cross section of the pipeline. These locations correspond to the vicinity of the acoustic source, on the central axis of the pipeline, and far away from the acoustic source.

The acoustic intensity measured near the acoustic source experiences the fastest delay along the axial direction, followed by the acoustic signal at the central axis and that far away from the acoustic source (e.g., the bottom end of the pipeline wall). This feature indicates that, if the leakage detection is conducted along the length direction of the pipeline, then an apparent acoustic intensity decay curve can be obtained near the acoustic source; contrarily, the same curve measured at a location far away from the acoustic source does not manifest any significant intensity variation. Therefore, effective leakage information cannot be extracted directly from the signal amplitude.

Owing to the standing wave effect in the tube, a decay curve with significant intensity change cannot be obtained at locations far away from the acoustic source. However, the spatial fluctuation of the acoustic intensity caused by the standing wave can be used for signal detection and analysis. The premise of using the standing wave effect to extract and analyse the leakage acoustic wave signal is that the frequency spectrum of the leakage acoustic source must exhibit a narrow distribution. If the acoustic source at the leakage point has a wide frequency spectrum, then more standing waves with different wavelengths will be generated, weakening the spatial fluctuations of the acoustic intensity.

### 4.2. Effect of Distribution of Monitoring Points

**Figure 5:**
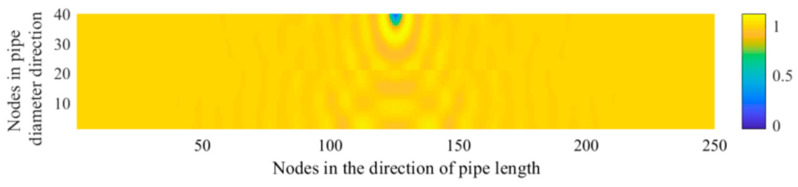
Distribution of the acoustic pressure generated from a point-shaped leakage hole along the longitudinal cross section of the 30-meter-long pipeline.

To analyse the acoustic characteristics detected at monitoring points with different distances from the acoustic source, the length of the model shown in Figure 1 was increased from 20 to 30 m and the corresponding computation region of the pipeline was extended to 30 m. By analysing the characteristics of the frequency spectrum at different monitoring points, we can evaluate the detection performance of the leakage acoustic wave generated at different locations. The monitoring points in the pipeline are distributed at these following locations: the 20th, 60th, and 120th nodes of the 250 nodes along the length direction of the pipeline. The exact locations of the monitoring points were set on the central axis below the acoustic source. Figure 5 shows the distribution of the acoustic pressure along the longitudinal section of the computational region in the extended pipeline. The superimposition of the reflected acoustic wave and the acoustic wave generated from the acoustic source can both be easily observed on the lower pipeline wall in Figure 5.

**Figure 6:**
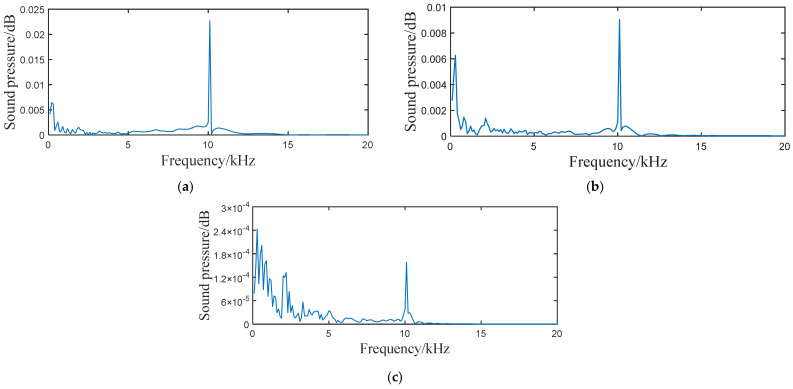
Acoustic pressure signal generated from a point-shaped leakage hole in the frequency domain measured at the (**a**) 20th, (**b**) 60th, and (**c**) 120th nodes along the direction of the water flow.

**Figure 7:**
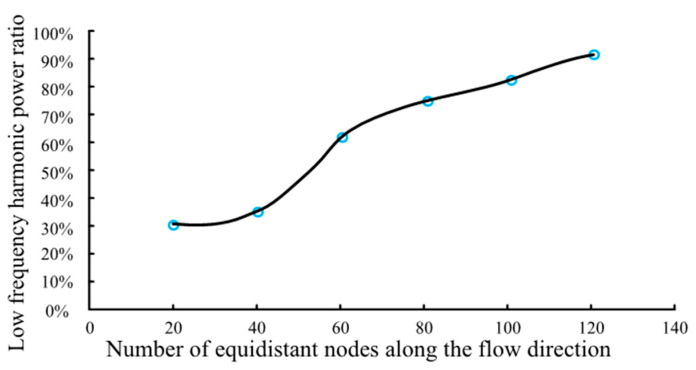
Proportion of the low-frequency harmonic power to the full-band power.

Figure 6 shows the acoustic pressure signals in the frequency domain at the 20th, 60th, and 120th nodes along the direction of the water flow. Figure 7 shows the proportion of the low-frequency harmonic power to the full-band power. It is found that the farther the monitoring point is from the sound source, the greater the proportion of the low-frequency harmonic power to the full-band power is. Based on our analysis, this is because the farther the monitoring point is from the sound source, the greater the change in the waveform of the acoustic source caused by reflection and superposition is. According to this phenomenon, when the leakage acoustic wave is detected along the length direction of the pipeline in the frequency domain, the signal is found to change from the superposition of low-frequency harmonics and the high-frequency waves of the acoustic source to high-frequency waves of a single acoustic source. Thus, if the detector is moving in the pipeline, the high-frequency signal is more evident when the detector is closer to the leakage point. Therefore, the location of the leakage point can be determined from the frequency spectrum of the signal, which is obtained using the detector.

### 4.3. Effect of Acoustic Source Frequency

**Figure 8:**
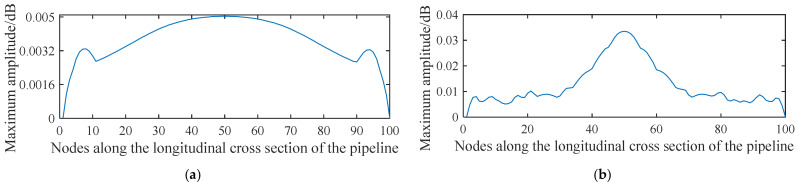
Distribution of the maximum amplitude of acoustic pressure generated from a point-shaped leakage hole along the longitudinal cross section of the pipeline for a source frequency of (**a**) 5000 Hz and (**b**) 10,000 Hz.

The propagation characteristics of acoustic sources with different frequencies in the pipeline are also important for the selection of the detector. In this section, we analysed the decay characteristics of acoustic sources with different frequencies propagating in the pipeline. Figure 8 shows the distribution of the maximum amplitude of the acoustic pressure in the longitudinal cross section of the pipeline for acoustic sources with frequencies of 5000 and 10,000 Hz. When the frequency of the acoustic source is 10,000 Hz, the acoustic pressure decays along the length direction of the pipeline is evident. Contrarily, when the frequency of the acoustic source is 5000 Hz, the decay curve of the acoustic pressure is relatively flat along the length direction of the tube. Therefore, the frequency of the acoustic source has a significant influence on the decay characteristics of the leakage signal in the pipeline.

### 4.4. Effect of Pipeline Dimension

**Figure 9:**
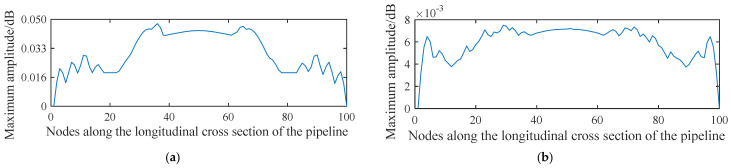
Distribution of the maximum amplitude of acoustic pressure generated from a point-shaped leakage hole along the longitudinal cross section of a pipeline with diameter of (**a**) 0.2 m and (**b**) 0.5 m.

To analyse the influence of different pipeline diameters on the decay characteristics and frequency spectrum, two different pipeline diameters were selected for calculation and analysis. Figure 9 shows the distribution of the maximum amplitude of acoustic pressure in the longitudinal cross section of pipelines with diameters of 0.2 and 0.5 m. As the pipeline diameter increases, the decay of the acoustic pressure intensity weakens along the length direction of the pipeline. In fact, the larger the pipeline diameter is, the weaker the characteristics of the low-frequency harmonic wave at the monitoring point are. This is because a larger pipeline diameter provides greater propagation space for the acoustic wave, which results in weaker reflection and superposition features. Consequently, the acoustic pressure intensity of the low-frequency harmonic wave becomes smaller.

### 4.5. Effect of Acoustic Source Geometry

The leakage points on underground water supply pipelines are usually point-shaped or small band-shaped cracks. These two types of cracks lead to point-shaped and long strip-shaped acoustic sources. A point-shaped acoustic source was selected and analysed in the previous sections. In this section, we analyse the characteristics of acoustic waves generated from a long strip-shaped acoustic source. The same velocity inlet and pressure outlet boundary conditions shown in Figure 1 were used for the analysis in this section. Ten computation nodes on the pipeline wall were selected as the acoustic source for generating sine waves. This configuration allows us to analyse the influence of the acoustic source distribution on the decay characteristics and frequency spectrum of the acoustic waves inside the pipeline.

**Figure 10:**
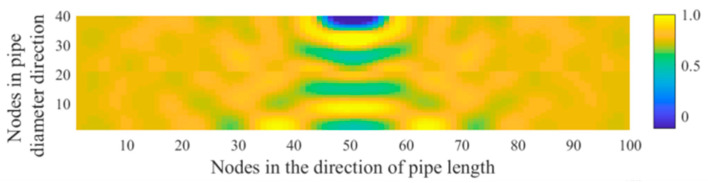
Distribution of the acoustic wave along the longitudinal cross section of the pipeline (long strip-shaped acoustic sources).

**Figure 11:**
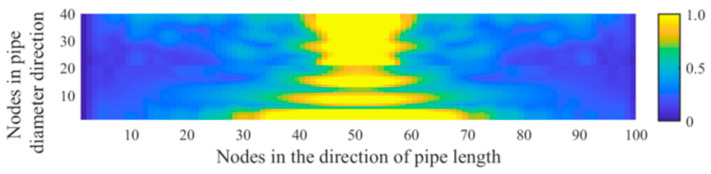
Maximum amplitude response curve of the acoustic pressure generated from a long strip-shaped leakage hole along the longitudinal cross section of the pipeline.

**Figure 12:**
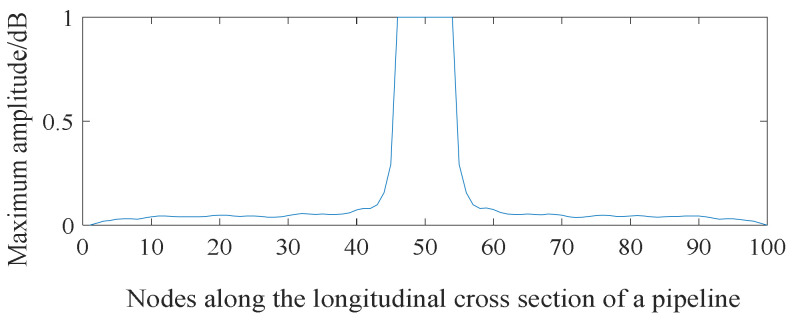
Distribution of the maximum amplitude of acoustic pressure generated from a strip-shaped leakage hole along the longitudinal cross section of a pipeline.

Figure 10 shows the distribution of the acoustic pressure on the longitudinal cross section of the pipeline. Figure 11 shows the distribution of the maximum amplitude of the acoustic pressure on the longitudinal cross section of the pipeline. The distribution pattern of the acoustic field intensity in the pipeline is more oriented along the radial direction of the pipeline. Almost no stripe pattern is observed along the length direction of the tube. This feature indicates that, when the acoustic source has a shape of a long stripe, the propagation direction of the standing wave in the pipeline is more inclined towards the radial direction of the pipeline. Theoretically, it can be concluded that, for a long strip-shaped acoustic source, it is not feasible to detect the leakage by measuring the spatial fluctuation of the acoustic wave caused by the standing wave along the flow direction. In addition, for long strip-shaped acoustic sources, the acoustic wave signals collected near the acoustic source inside the pipeline during the movement along the length direction of the pipeline show a ‘single flat peak’ feature in the response amplitude, as shown in Figure 12. Further, unlike the single point acoustic source shown in Figure 4, the long strip-shaped acoustic source only generates a very small spatial fluctuation of the acoustic intensity along the flow direction. This is because the waves of long strip-shaped acoustic sources mostly propagate along the radial direction of the pipeline. Therefore, the reflected waves will also propagate along the radial direction. Thus, the standing waves generated by the superimposition of these two waves are mostly distributed along the radial direction. The difference between the point-shaped and long strip-shaped leakage hole can be utilised for leakage hole recognition. The signal from the long strip-shaped leakage hole, along the radial direction, is more obvious than that from the point-shaped hole. Furthermore, the ‘single flat peak’ can be used as another identification characteristic.

## 5. Experimental Study

### 5.1. Experimental Setup

**Figure 13:**
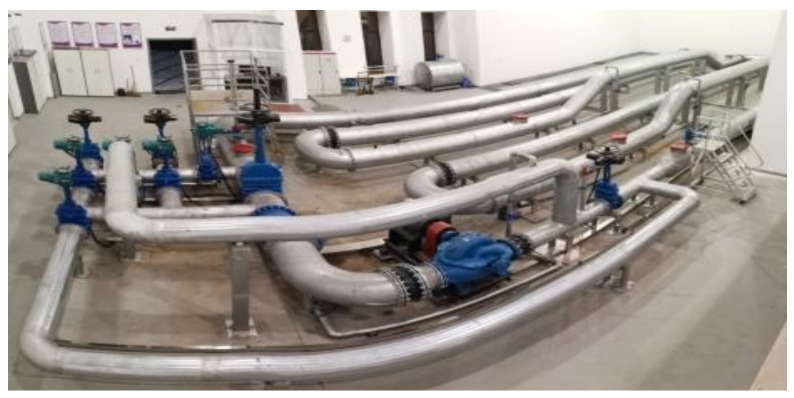
Actual water supply pipeline used in the simulated leakage experiment.

A self-designed experiment platform that simulates the leakage in a pipeline was constructed according to the actual scale of a water supply pipeline [24]. A self-developed mobile spherical detector for use in the water supply pipeline [25,26] was employed to collect the leakage acoustic signal generated from simulated leaking holes located at different distances and directions with respect to the pipeline. The actual water supply pipeline used in the simulated leakage experiment is shown in Figure 13.

**Figure 14:**
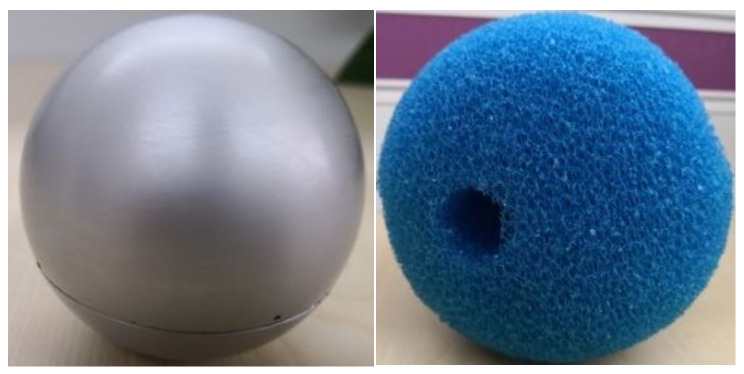
Mobile spherical detector for use in the water supply pipeline.

The mobile spherical detector for use in the water supply pipeline is composed of an inner core metal ball and an outer sponge spherical shell. Images of the physical objects are shown in Figure 14. The core metal ball is responsible for the collection and storage of the leak acoustic wave data. In the actual detection process, the core metal ball is placed inside the sponge spherical shell to provide good vibration damping. In recent years, microphones have gained popularity in detecting faults owing to their high sensitivity and bandwidth [27]. Therefore, we have used a bone conduction microphone sensor as the acoustic sensor in this study.

**Figure 15:**
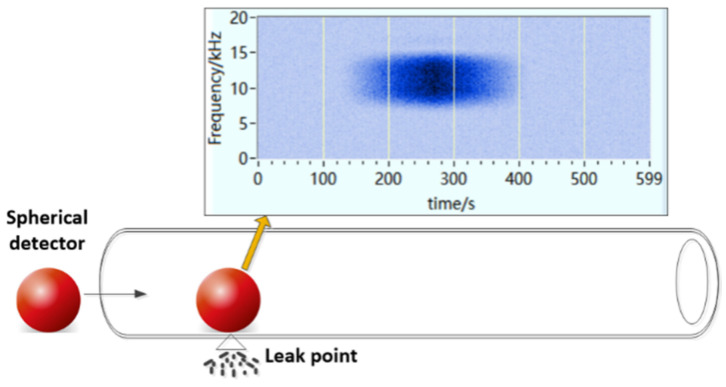
Working mode of the mobile spherical detector.

The working mode of the aforementioned mobile spherical detector is shown in Figure 15. The spherical detector moves smoothly in the pipeline with the help of the fluid thrust in the pipeline. Upon passing a leakage point in the pipeline, the built-in high-sensitivity acoustic sensor captures the acoustic wave generated by the leakage in the pipeline. Simultaneously, the built-in accelerometer is used together with the ground marker to locate the leakage point accurately. Such an approach allows us to detect leakage in the pipeline.

**Figure 16:**
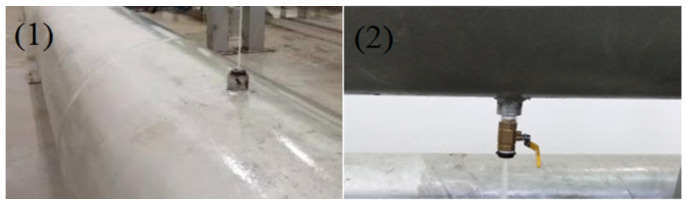
Simulated leakage hole on the (1) upper and (2) lower surfaces of the pipeline.

To analyse the characteristics and propagation behaviour of the leakage acoustic signal in water supply pipelines under different conditions, simulated experiments were conducted using pipelines with two different diameters (0.2 and 0.5 m). The shape of the leak holes includes both a circular point and a long strip. In our experiment, simulated leak holes were designed at the following two different locations, as shown in Figure 16.

### 5.2. Analysis of Experimental Results

**Figure 17:**
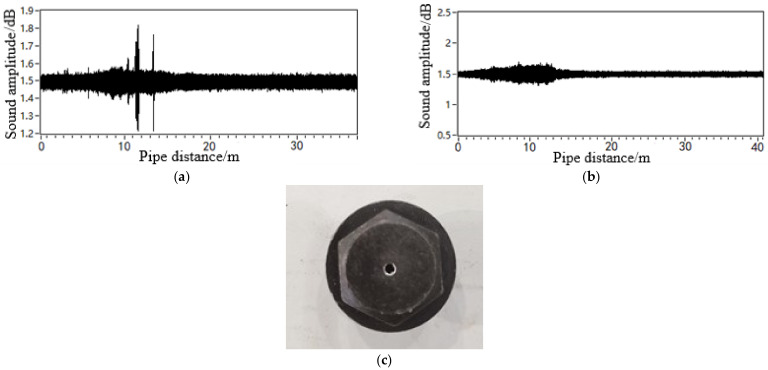
Leak acoustic signal generated from a point-shaped leakage hole in a pipeline with a diameter of 0.5 m located on the (**a**) lower and (**b**) upper surfaces of the pipeline; (**c**) the leakage hole geometry of point circle.

Figure 17 shows the waveform of the leakage acoustic wave generated by leakage on the upper and lower surfaces of a pipeline with a diameter of 0.5 m and the leakage hole geometry of the point circle. The data were collected using the spherical detector. As shown in Figure 17, the spherical detector moves on the bottom surface of the pipeline during the detection process. Therefore, the detector is closer to the leakage acoustic source on the lower surface than that on the upper surface of the pipeline. A comparison of the waveform associated with the two leakage locations demonstrated a significant decaying effect of the acoustic intensity, which is consistent with the simulation results discussed in Section 4.1.

**Figure 18:**
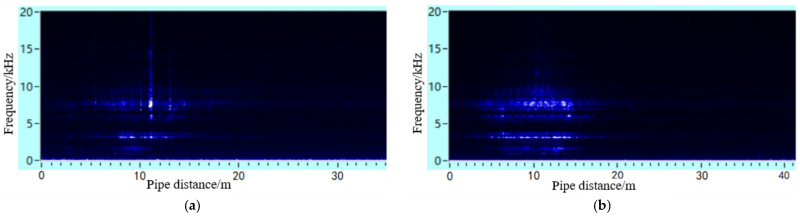
Time-frequency diagram of leakage acoustic signal generated from a circular point hole in a pipeline with a diameter of 0.5 m on the (**a**) lower and (**b**) upper surfaces of the pipeline.

**Figure 19:**
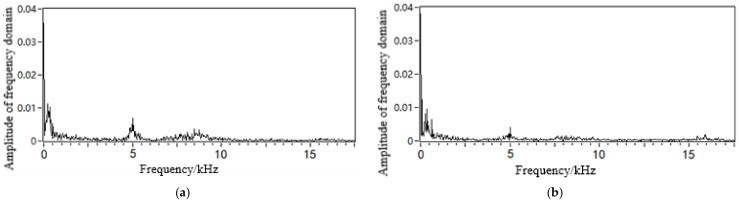
Frequency spectrum of the leakage acoustic signal generated from a circular point hole in a pipeline with a diameter of 0.5 m collected (**a**) 5 m and (**b**) 10 m away from the leakage hole.

The leakage acoustic wave signal can be analysed using short-time Fourier transform (STFT) calculations to obtain the time-frequency analysis diagram, as shown in Figure 18. It can be seen that whether the leakage hole is located on the upper or lower surface, a richer number of high-frequency components can always be found in the acoustic wave signal at locations closer to the leakage hole. At locations farther from the leakage hole, the high-frequency component experiences a fast decay, while the low-frequency component attenuates slowly. A frequency spectrum analysis was further performed on the acoustic signals collected at distances of 5 and 10 m away from the leakage hole, as shown in Figure 19. These experimental data results are in accordance with the trends of the simulation analysis results discussed in Section 4.2 and Section 4.3.

**Figure 20:**
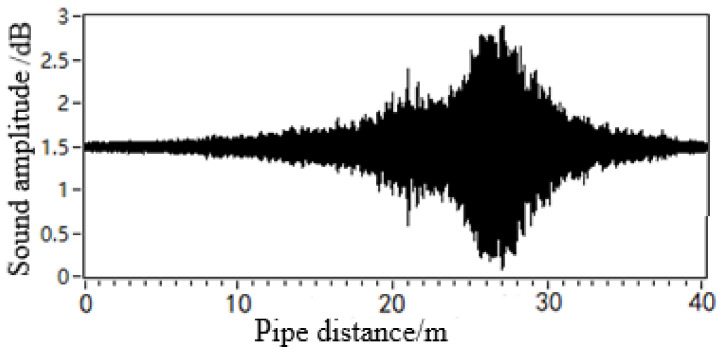
Leakage acoustic signal generated from a circular point hole in a pipeline with a diameter of 0.2 m.

**Figure 21:**
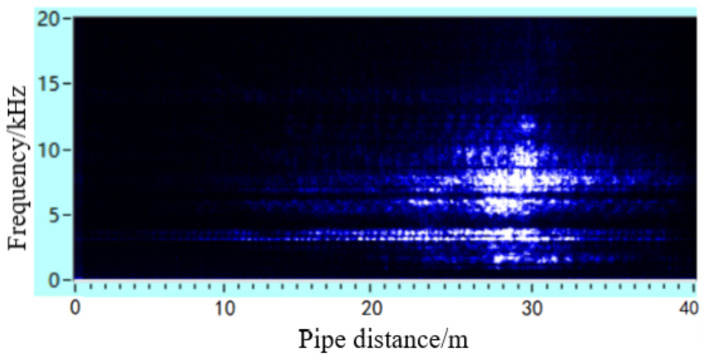
Time-frequency diagram of the leakage acoustic signal generated from a circular point hole in a pipeline with a diameter of 0.2 m.

All the experimental data were obtained on the basis of the leakage acoustic wave data collected from a pipeline with a diameter of 0.5 m. To analyse the characteristics of the leakage acoustic wave under different pipeline diameter conditions, the acoustic wave signals generated from the leakage hole were collected by the spherical detector in a pipeline with a diameter of 0.2 m, as shown in Figure 20. Moreover, there is a circular point leakage hole on the upper surface of the pipeline. The experimental conditions pertaining to the signals in Figure 17b and Figure 20 are different in terms of the diameter. The acoustic pressure intensity of the acoustic wave signal shown in Figure 20 was found to be higher than that shown in Figure 17b when approaching the leakage point. However, the decay of the signal also becomes more significant. After performing STFT calculations on the signal data shown in Figure 20, we obtained the time-frequency analysis diagram shown in Figure 21. A comparison with the results shown in Figure 18b suggests that the low-frequency component in the leakage acoustic signal collected in the pipeline with a diameter of 0.2 m decays faster along the length direction of the pipeline. This feature is consistent with the trends of the simulation results described in Section 4.4.

**Figure 22:**
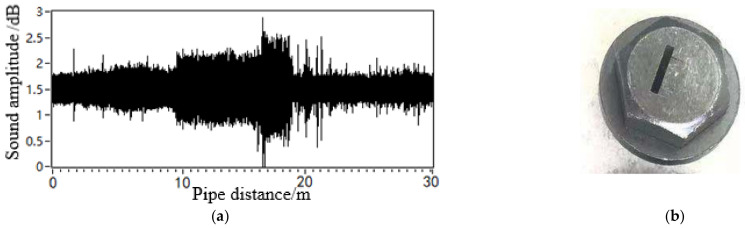
Leakage acoustic signal generated from a long strip-shaped hole in a pipeline with a diameter of 0.5 m: (**a**) leakage acoustic signal, (**b**) geometry of the long strip-shaped hole.

**Figure 23:**
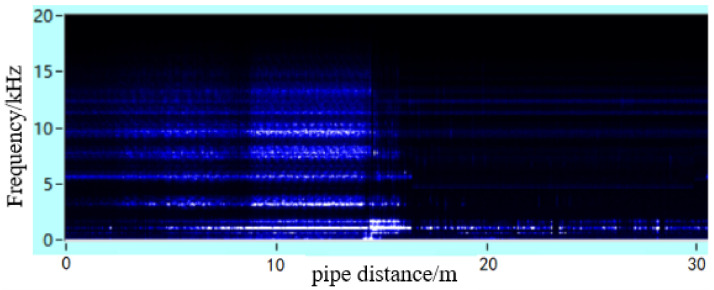
Time-frequency diagram of the leakage acoustic signal generated from a long strip-shaped hole in a pipeline with a diameter of 0.5 m.

To analyse the effect of the geometry of the leakage hole in the pipeline on the propagation behaviour of the leakage acoustic wave, the spherical detector was further used to collect the leakage acoustic wave signal generated from a long strip-shaped leakage hole in the pipeline, as shown in Figure 22. The acoustic wave signal collected near the acoustic source inside the pipeline during the movement along the length direction of the pipeline exhibits a significant ‘single flat peak’ feature. Figure 23 shows the time-frequency diagram obtained from STFT calculations. The energy of the signal is concentrated on multiple frequency components at unequal intervals because the leakage acoustic wave propagating in the pipeline superimposes with the reflected wave along the radial direction and generates standing waves when the leakage hole is long strip-shaped. This finding is consistent with the simulation analysis results discussed in Section 4.5.

## 6. Conclusions

In this study, a 3D transient leakage acoustic propagation model in a water supply pipeline was developed by combining the fundamental principles of fluid dynamics with Lighthill acoustic analogy theory. Through simulation analysis, we analysed the distribution of the acoustic pressure and acoustic intensity in the pipeline. The effect of different factors, such as the pipeline size and characteristics of the acoustic source, on the propagation of a leakage acoustic wave was also investigated in this study. Finally, experiments were performed to validate the simulation results. The following conclusions were drawn:

(1) The acoustic waves reflected by the lower surface of the pipeline and those transmitted from the acoustic source are superimposed in the centre of the pipeline to form interference fringes. The acoustic intensity is greater near the acoustic source in the pipeline and decays quickly when moving away from the acoustic source. When performing leakage detection along the length direction of the pipeline, a significant decay curve of the acoustic intensity can be obtained near the acoustic source. However, the shape of the acoustic wave becomes planar at locations far away from the acoustic source. Meanwhile, the acoustic intensity does not decay significantly at locations far away from the acoustic source.
(2) As the pipeline diameter increases, the decay of the acoustic pressure intensity along the length direction of the pipeline becomes weaker, and the dispersion characteristics become less evident. When the leakage acoustic wave generated by the long strip-shaped leakage hole propagates in the pipeline, it is superimposed with the reflected wave in the radial direction of the pipeline to produce a propagating standing wave. The superimposed wave exhibits a significant ‘single flat peak’ feature in the response amplitude along the length direction of the pipeline.
(3) The farther the monitoring point is from the acoustic source, the greater the proportion of the low-frequency harmonic acoustic pressure intensity is. During the detection of the leakage acoustic wave along the length direction of the pipeline, the signal changes from the superposition of low-frequency harmonics and high-frequency waves of the acoustic source to high-frequency waves of a single acoustic source. In addition, the high-frequency component in the leakage acoustic wave decays more substantially than the low-frequency component along the length direction of the pipeline. As shown by the simulation analysis and simulated experiments, the leakage acoustic wave signal experiences a significant frequency shift along the length direction of the pipeline. Therefore, comprehensive analysis of the characteristics in both the low- and high-frequency ranges is required to detect leakage in water supply pipelines.
